# Impact of Immunization Technology and Assay Application on Antibody Performance – A Systematic Comparative Evaluation

**DOI:** 10.1371/journal.pone.0028718

**Published:** 2011-12-20

**Authors:** Michael C. Brown, Tony R. Joaquim, Ross Chambers, Dale V. Onisk, Fenglin Yin, Janet M. Moriango, Yichun Xu, David A. Fancy, Erin L. Crowgey, Yida He, James W. Stave, Klaus Lindpaintner

**Affiliations:** 1 Research and Development, SDIX, Newark, Delaware, United States of America; 2 Antigen Design Laboratory, SDIX, Newark, Delaware, United States of America; 3 BioProcessing Department, SDIX, Newark, Delaware, United States of America; King's College London, United Kingdom

## Abstract

Antibodies are quintessential affinity reagents for the investigation and determination of a protein's expression patterns, localization, quantitation, modifications, purification, and functional understanding. Antibodies are typically used in techniques such as Western blot, immunohistochemistry (IHC), and enzyme-linked immunosorbent assays (ELISA), among others. The methods employed to generate antibodies can have a profound impact on their success in any of these applications. We raised antibodies against 10 serum proteins using 3 immunization methods: peptide antigens (3 per protein), DNA prime/protein fragment-boost (“DNA immunization”; 3 per protein), and full length protein. Antibodies thus generated were systematically evaluated using several different assay technologies (ELISA, IHC, and Western blot). Antibodies raised against peptides worked predominantly in applications where the target protein was denatured (57% success in Western blot, 66% success in immunohistochemistry), although 37% of the antibodies thus generated did not work in any of these applications. In contrast, antibodies produced by DNA immunization performed well against both denatured and native targets with a high level of success: 93% success in Western blots, 100% success in immunohistochemistry, and 79% success in ELISA. Importantly, success in one assay method was not predictive of success in another. Immunization with full length protein consistently yielded the best results; however, this method is not typically available for new targets, due to the difficulty of generating full length protein. We conclude that DNA immunization strategies which are not encumbered by the limitations of efficacy (peptides) or requirements for full length proteins can be quite successful, particularly when multiple constructs for each protein are used.

## Introduction

The post-genomic era has ignited a growing demand for the cost-effective generation of high quality, affinity-purified polyclonal reagents to support the routine detection and/or measurement of numerous protein biomarkers in basic and applied research, and as diagnostic tools. Antibody reagents support traditional immunodetection tools such as immunoblotting, immunohistochemical (IHC) analysis, immunoprecipitation, flow cytometry, ELISA, as well as more advanced proteomic assay platforms such as planar or bead-based antibody multiplexing microarrays and antibody-oriented mass-spectrometry technologies [1]–[7]. In designing immunization strategies for these immunodetection methods, the epitope on the target protein that is recognized by the antibody can exist in multiple conformations, ranging from linear, as in a fully denatured protein, to conformationally complex epitopes that are more rigidly structured and often composed of several discontinuous regions, as displayed in folded proteins [8].

The generation of antibody reagents to meet the demands of proteomic applications continues to be driven by conventional protein immunization approaches [9]. Classical protein immunization strategies most often rely on synthetic peptides [6], [7], [10], [11], large fragment or full-length recombinant proteins of bacterial [9], [12], [13] or mammalian cell origin [6], or purified native proteins [14] as sources of immunogens.

By virtue of their low cost, simplicity of synthesis, and historical track-record for polyclonal and monoclonal antibody production the use of peptides as immunogens is widespread [6], [10], [11], [15], [16]. Antibodies raised against peptides represent the majority of antibodies available through antibody catalog vendors. Because very small peptides are poorly immunogenic [14] and large ones are challenging to synthesize, peptide fragments deployed as immunogens typically consist of 12 to 20 amino acid residues [6], [10], [11], [15]. A number of limitations are not always appreciated constrain the utility of peptide immunizations. Among them are challenges in antigen design based on issues such as lack of effective algorithms for predicting surface regions in the absence of protein structure information or B cell epitopes and [17]–[19]. Moreover, the conventionally used size of 12–20 residues rarely encompasses more than a single epitope and is likely to lack secondary and tertiary conformational structure [9], [10].Consequently, it is much less likely to generate antibodies capable of binding natively folded protein [8] in a sandwich ELISA, although they can work well in many applications against the protein in a denatured form and have widespread proteomic applications.

Full length protein antigens address many of the limitations attributed to peptides. Inherently, they contain surface regions, multiple immunogenic epitopes, and are likely to fold to form (at least partially) native structures even if synthesized in prokaryotic systems [9]. However, recombinant synthesis and/or purification of full length protein antigens can be a daunting task, takes significant time and resources, and is encumbered by uncertainty regarding successful production [20].

More innovative approaches such as DNA (or “genetic”) immunization have emerged as alternative and/or complementary tools to classical antibody generation strategies. DNA immunization employs an expression plasmid encoding the selected antigen to immunize animals. The transfected tissues of the immunized animal express the antigen which subsequently drives an antibody response [21]–[25]. DNA immunization with sequences coding polypeptide protein regions combines the advantages of both full length protein and peptide and immunization approaches, providing immunogens that comprise relatively large regions of the target protein with the potential for multiple epitopes, faster turn-around, and greater accessibility than full-length protein. We chose a strategy of using DNA for priming, and using the protein fragment encoded by the DNA-construct for boosting. It has been consistently reported that this combination strategy results in clearly superior antibody responses when compared to immunizations using either DNA for both priming and boosting, or a protein fragment for both priming and boosting. [26]–[28]. These published reports are entirely in keeping with our own in-house observations and experience.

Despite the widespread use of synthetic peptide antigens for generating antibodies, no well controlled, quantitative, directly comparative studies demonstrating their performance on multiple targets relative to other antigens such as purified protein have been published. Most reports deal with a single protein in a limited number of applications. Many reports discuss the performance of off-the-shelf reagents obtained from catalog vendors; however, they generally relate anecdotal evidence and their interpretation is often limited due to lack of information on study design controlling for any of the many variables that influence antibody production and immunoassay performance (antigen design, immunization protocols, immunoassay variables) [29]–[33].

To better understand the strengths and weaknesses of different immunization approaches we carried out a systematic study comparing 3 immunization strategies, i.e., using peptides (Pep-Abs), DNA (DNA-Abs; i.e. DNA prime/encoded polypeptide boost), and full length protein (FLP-Abs) to generate antibodies against 10 different serum proteins. These were selected based on their commercial availability as high purity, native full length proteins. These proteins are well established markers in clinical medicine and the steps that led them there, including generation of high quality antibodies, could serve as models for newly discovered biomarkers. The polyclonal antibodies against these targets made by the 3 immunization strategies were then stringently evaluated for fitness of use against their target full-length protein in several commonly used immunologic techniques, including those where the protein exists in a relatively native state, such as ELISA, and those in which the target protein exists in different states of denaturation, such as Western blots and IHC.

## Methods

### Antigens

Proteins targeted in this study were transferrin (TF), thyroglobulin (TG), thyroxine binding globulin (TBG), alpha-fetoprotein (AFP), sex hormone binding globulin (SHBG), prostate specific antigen (PSA), carcinoembryonic antigen (CEA), alpha-1-antitrypsin (AAT), alpha-2-macroglobulin (A2M), and prostatic acid phosphatase (PAP). Proteins were obtained from SCIPAC (Sittingboure, Kent, UK) and Lee Biosolutions (St. Louis, MO). Purity was analyzed by SDS PAGE and was greater than 98% for all proteins. All proteins were stored at −80 degrees C unless otherwise recommended by the manufacturer.

### Immunogen Design and Synthesis

For each of the 10 target proteins non-overlapping regions were selected as peptide antigens. The peptides were designed and synthesized by three leading suppliers; 21^st^ Century (Marlboro, MA), Pi Proteomics (Huntsville, AL), and New England Peptide (Gardner, MA). The peptide suppliers used their in house methods to generate the designs and up to 3 designs were provided from each company for each target, resulting in a total of 68 individual peptide designs to the 10 targets. One non-overlapping design was then selected from each company for each target, giving a total of 30 peptide designs. The 30 designs were selected from the pool of 68 designs using the following additional criteria: designs were ranked based on surface accessibility (we argued that peptides buried in the folded protein are unlikely to yield antibodies that bind native, folded protein in a sandwich ELISA), the absence of any post-translational modification (since the peptide immunogen will not contain these), and lastly sequence identity to paralogs to ensure that the antibodies are specific. Structural information was available for most of the 10 targets and encompassed the regions for 58 of the peptide designs. Upon review, 15 peptide designs received for the 3 companies solicited were rejected due to lack of surface accessibility in the structure, and 5 were rejected because of identity to a paralog, or because they contained sites of post-translational modification. The remaining peptides were ranked by their identity to the host animal with the lowest identity selected to avoid immune tolerance. The peptide lengths varied from 10 to 20 residues, with an average length of 16 residues. Peptides were synthesized with a cysteine at either the N- or C-terminus of the peptide to allow conjugation. Peptides were >85% pure and conjugated to KLH using maleimide chemistry (Pierce, Rockford, IL) for immunization.

**Figure 1:**
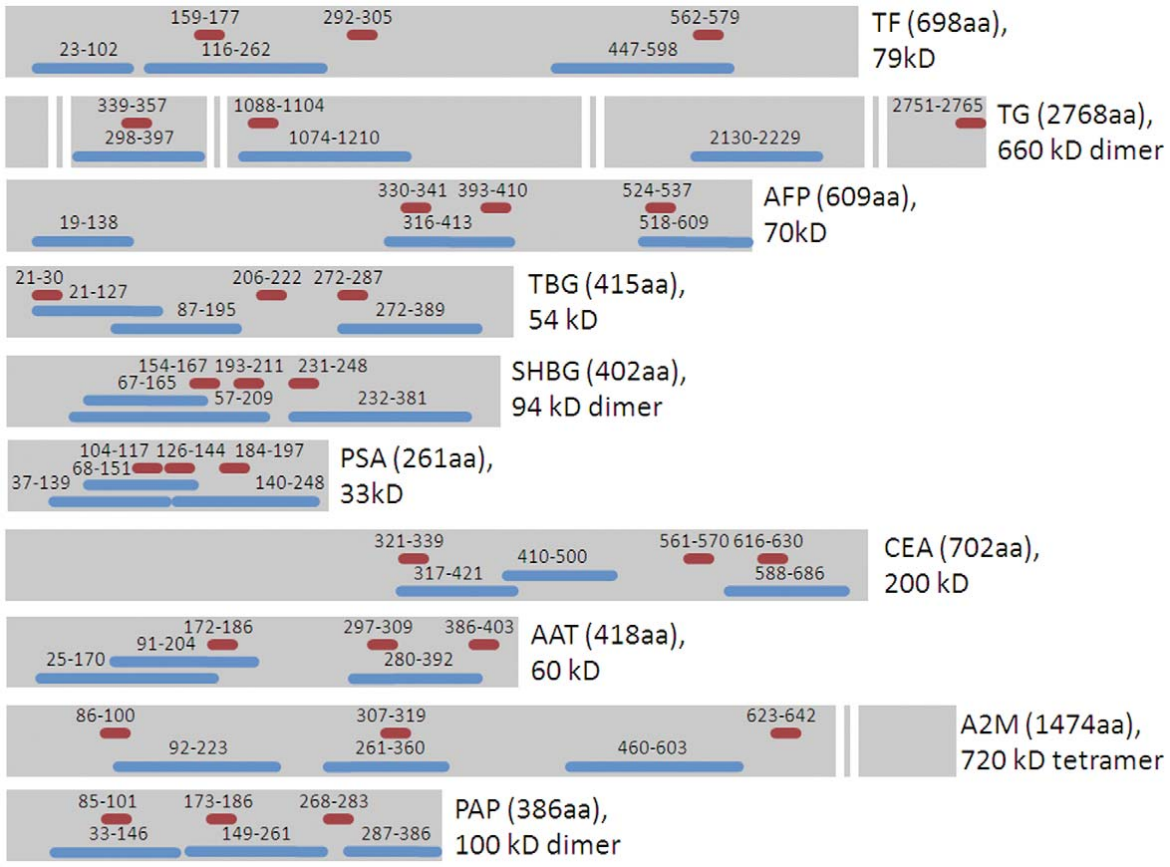
Regions of proteins selected for peptide and DNA immunization designs. Each protein is represented in grey with the length in amino acids of the monomeric unit indicated. 2 proteins, TG and A2M are too large to fit proportionally and gaps in the grey band are shown in areas where no designs were selected. Designs selected for peptides are shown in red and designs for DNA shown in blue.

Three DNA immunization antigens per target were designed according to similar principles to those followed in the peptide design, although regions were primarily selected to encompass an entire domain, or a compact folded region. Domains were selected based on Uniprot listed domain information. Structural information was not available for 5 of the 30 designs. Surface accessibility was not relevant for ranking since all encompassed large regions of the protein, (80–152 residues, average 114), and thus all the designs contained substantial surface exposed regions. Sequences were ranked and selected based on lowest identity to the host animal, lowest identity to paralogs, and avoidance of post-translational modifications. Synthetic genes of the antigens were synthesized (Integrated DNA Technology, Coralville, IA) and cloned into a DNA immunization plasmid based on pCI (Promega, Madison, WI) and an *E. coli* expression plasmid based on pSP2b (Sigma, St. Louis,MO) with a Ptac-lac promoter. The latter plasmid was used to produce the portion of the protein encoded by the DNA construct for an antibody affinity purification column and for a single protein fragment boost [26]–[28] following DNA immunization to improve antibody yields. This *E.coli* protein, containing a his tag, was produced in cultures induced by ITPG, spun down, frozen, and lysed, Lysate was solubilized in 7 M Guanidine and purified over HisPur Cobalt resin (Thermo Fisher Scientific, Rockford, IL) using 250 mM imidazole for elution. Sequence details of peptide and DNA designs are shown in Figure 1.

### Immunization

All animal work was approved prior to start by the SDIX Institutional Animal Care and Use Committee and performed in a USDA Registered (50-R-0013), AAALAC accredited (accreditation number 001011) facility. In total 140 rabbits were used in this study. Immunization regimes were consistent with well-established industry protocols [34]. Two New Zealand white rabbits, 12 weeks of age, weighing 5.5 to 6.5 lbs, were immunized with each of the antigens (30 peptides, 30 DNA constructs, 10 full-length proteins). KLH conjugated peptides (0.2 mg per rabbit) were administered in complete Freund's adjuvant (CFA) by subcutaneous injection of 4 different sites. At weeks 3, 5 and 7 animals were boosted with 0.2 mg of conjugated peptide in incomplete Freund's adjuvant (IFA) administered as described previously. Serum was collected over a 3 day period 2 weeks after the final boost. The DNA antigen constructs were conjugated to nano-gold particles and administered via gene gun (Helios, Biorad, Hercules,CA) to the inside of the ears (3 µg total) and boosted with DNA (gene gun) at week 2 (3 ug). At week 5 animals were given a single boost of 100 µg of recombinant *E. coli* protein fragment derived from the same gene fragment in CFA by subcutaneous injection of 4 different sites, and serum was collected at week 7. Full length protein (250 µg per rabbit) was administered following the same immunization routes and schedule as described for peptides.

### Antibody Purification

To minimize the effect of animal-to-animal variability the sera from each set of 2 rabbits were pooled for purification and analysis. Sera from each treatment group were purified on their respective immunogen . Pep-Abs were affinity-purified using the cognate peptide used for immunization coupled via the N- or C-terminal cysteine to sepharose [35]. DNA Abs were purified on column resins to which we coupled the recombinant *E. coli* protein fragment derived from the same sequence used for the DNA immunization. FLP-Abs were were affinity purified on the purified, full length serum proteins coupled to cyanogen-bromide activated sepharose (GE, Piscataway, NJ). Antibodies were eluted using 0.1 M glycine buffer, pH 2.5, neutralized immediately and dialyzed against PBS for a total of 3 buffer exchanges. Purified antibodies were quantified by absorbance at 280 nm. For sandwich assays, antibodies were biotinylated using NHS-LC- Biotin (Pierce, Rockford, IL).

### Direct Bind ELISA

Direct bind ELISA was performed with purified full-length antigen coated at a concentration of 1 ug/ml in 0.05 M carbonate buffer pH 9.6 dispensed at 100 µl per well in 96-well microplates (Nunc MaxiSorp, Nalge Nunc, Rochester, NY) [33]. Three-fold serial dilutions of antisera were evaluated using standard protocols and developed with goat-anti-rabbit IgG, Fc specific (Jackson Immunochemicals, West Grove PA) secondary antibody and tetramethylbenzidine/peroxide solution (TMB, Moss, Pasadena, MD) as the color developing reagent. Plates were read after 15 minutes of development in a microtiter plate reader (SpectraMax, Molecular Devices, Sunnyvale, CA).

### Sandwich ELISAs

Sandwich immunoassays were performed by coating purified antibodies at a concentration of 2.5 ug/ml in 0.05 M carbonate buffer pH 9.6, 100 µl per well onto 96 well microplates (0.25 µg of antibody per well). Four-fold serial dilutions of purified full-length native antigen ranging from 100 ng/ml to 0.024 ng/ml were added to the wells, followed by subsequent addition of biotinylated purified antibody (0.5 ug/ml), and horseradish peroxidase labeled streptavidin (0.2 ug/ml; Jackson Immunochemicals, West Grove, PA). Color was developed as previously described. All antibodies within a treatment group for a protein (such as all Pep-Abs to a given target) were paired with each other, both as capture and detection antibodies. Additionally, FLP-Abs were paired with every Pep-Ab and DNA-Ab to that protein as both capture and detection antibodies. This resulted in a total of 31 different sandwich assays for each target.

### Western Blot Analysis

The immunological reactivity of 69 antibodies (one DNA-Ab was lost due to insufficient yield) derived from synthetic peptide, full-length protein, and DNA-polypeptide immunizations was characterized by Western blot analysis against denatured full-length protein samples. Protein samples were electrophoretically separated under denaturing conditions on SDS-PAGE, 4–20% Tris-HCl pre-cast gels in a Criterion Cell apparatus (Bio-Rad, Hercules, CA) in running buffer as recommended by the manufacturer (Bio-Rad, Hercules, CA). Fractionated proteins were electro-blotted onto nitrocellulose membranes (0.45 µm pore size, Protran, Whatman, Germany) in a semi-dry electrophoretic transfer cell unit (Trans-Blot® SD, Bio-Rad, CA) and then blocked with TBST (Sigma, St.Louis, MO) supplemented with 2% Difco skim milk (Becton Dickinson, Sparks, MD).

After an overnight incubation at 4–8°C, secondary antibody (horseradish peroxidase-conjugated goat anti-rabbit, Invitrogen, Carlsbad, CA) was added to each blot. Membrane blots were then developed with chemiluminescent substrate (SuperSignal® West Femto, Thermo Scientific, Rockford, IL) and the signal was captured with a multi-purpose Image Station 440CF system (ver. 3.6, Eastman Kodak, NY). Each immunoblot was inspected thoroughly for the presence of a band at the protein's expected molecular weight by adjusting brightness, contrast, and magnification settings using the 1D Image Analysis software (Kodak, ver. 1.0).

### Immunohistochemistry (IHC)

Immunohistochemical analysis on formalin-fixed, paraffin-embedded human tissue specimens was carried out, for reasons of cost and practicability, only using the antibodies raised against CEA and PSA, and was performed by LifeSpan BioSciences (Seattle, Washington). Following formalin-fixation, tissue specimens were de-paraffinized in xylene and rehydrated sequentially in a stepwise fashion with decreasing ethanol concentrations and a final wash in water.

After probing with anti-CEA and anti-PSA unlabeled primary antibody at dilutions ranging from 2.5 µg/ml to 20 µg/ml to allow for optimization , immunoreactivity to target proteins was detected with secondary biotinylated anti-rabbit IgG (Vector Laboratories, Burlingame, CA) and avidin-biotin-alkaline phosphatase complex (Vectastain ABC kit, Vector Laboratories, Burlingame, CA), visualized with Vector Red chromogen (Vector Laboratories, Burlingame, CA), and then counterstained with Harris hematoxylin (Richard Allan Scientific, Kalamazoo, MI). Stained slides were imaged with a DVC1310C digital camera mounted on a microscope (Nikon Eclipse E400, Nikon Instruments, Melville, NY). The degree of staining was assessed by a single pathologist blinded to the experimental variables of the study. Each antibody was then ranked on a scale of 1 to 7 where a rank of 1 was the highest-ranking performance in IHC using 3 criteria: specific staining of target tissues; least number of cells stained; and differential staining between target and control tissues.

### Data Analysis

Two-way analysis of variance (ANOVA) and a Welsh's t-test were computed for comparisons of means of antisera titers, antibody yields, antibody specific activities (defined as the amount of antibody required to produce a signal intensity of 0.5 absorbance units in direct bind ELISA), and for homologous and heterologous sandwich assay performance for antibodies generated by the 3 immunization strategies.

Odds ratios and their 95%confidence intervals (CI) were calculated for Western blot data and analyzed by Fisher's Exact Test using GraphPad Prism 5 (ver. 5.04; GraphPad Software, La Jolla, CA).

## Results

Antibodies were evaluated based on yield after affinity purification, specific activity of the purified antibody, and the ability of these antibodies to work in sandwich ELISAs, Western blots, and IHC. Affinity purification of the antibodies, each on the antigen used for immunization (in the case of the DNA-immunization group, the protein fragment encoded by the respective DNA sequence), allowed assays to be normalized and compared based on mass of antibody rather than just serum titer. Furthermore, the sandwich assays required the use of purified antibody for both the capture phase as well as for labeling as a detector. Thirty antibodies made to peptides (Pep-Abs), 29 antibodies made to polypeptides by DNA immunization (DNA-Abs; a very low yield occurred for 1 of the 30 immunizations) and 10 antibodies made to full length protein (FLP-Abs) were evaluated. These represented sera from 140 rabbits, two rabbits per treatment group.

**Figure 2:**
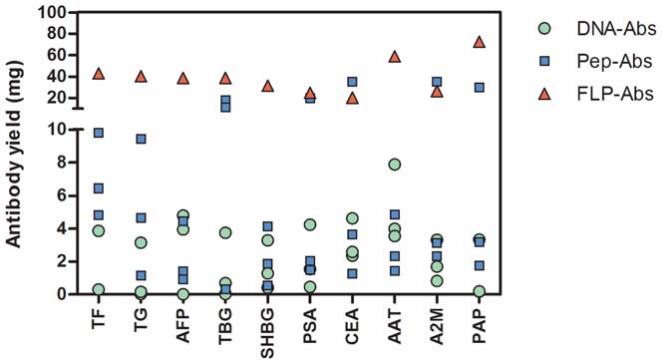
Yields of purified antibody following affinity purification from 80 ml of pooled rabbit sera. Each symbol represents the yield for each pool of 2 rabbits. There are 3 constructs per protein for each of the DNA-Ab and Pep-Ab methods and a single data point for the pool from full length native immunization (FLP-Abs).

Yields of antibodies after affinity purification varied widely (Figure 2). Purified antibody yields from FLP-Ab (mean for 10 proteins of 36.9 mg from 80 ml of antisera, standard deviation ±16.0 mg) were significantly higher (P<0.01) than yields using other immunization methods (Table 1). While there was high variability from protein to protein, Pep-Abs (geometric mean of 3.7 mg from 80 ml of sera) had significantly greater (P<0.01) yields than DNA-Abs (geometric mean of 0.8 mg).

**Table 1:** The overall effect of the various immunization strategies across ten different selected serum proteins on antibody yield, antisera titer, antibody specific activity, and assay sensitivity of different antibody combinations by ELISA.

					Sensitivity of Sandwich Assay (nM)^1^		
Immunization Method	Number of Antibodies	Yield^2^ (mg)	Specific Activity of Affinity Purified Antibody (ng/mL)	Antisera Titer^3^ (×10^5^)	Antibody Combinations Against:		
					Self	Native	Within Method
DNA-Abs	30	0.8	1	0.9	5.8×10^5^	4.9	3.3×10^5^
Pep-Abs	30	3.7	7.6	1.4	no detection	1.2×10^7^	no detection
FLP-Abs	10	36.9	0.1	109.7	1.2×10^−3^	1.2×10^−3^	1.2×10^−3^
		**p-value of Welsh two sample t-test**					
DNA-Abs vs. Pep-Abs		6.8×10^−04^	2.9×10^−04^	0.57	7.0×10^−04^	1.5×10^−07^	7.1×10^−04^
DNA-Abs vs. FLP-Abs		2.4×10^−11^	1.5×10^−04^	4.3×10^−10^	3.0×10^−11^	1.1×10^−04^	3.5×10^−10^
Pep-Abs vs. FLP-Abs		4.6×10^−11^	2.9×10^−09^	1.3×10^−10^	8.0×10^−13^	6.1×10^−15^	8.0×10^−13^

1 Geometric mean of assay sensitivity reported for homologous (self) and heterologous antibody combinations against native, and “within” immunization method.

2 Geometric mean of antibody yield for a 80-ml antiserum affinity purification run.

3 Geometric mean of antisera titers was intrapolated from a titration curve at 0.500 absorbance.

**Figure 3:**
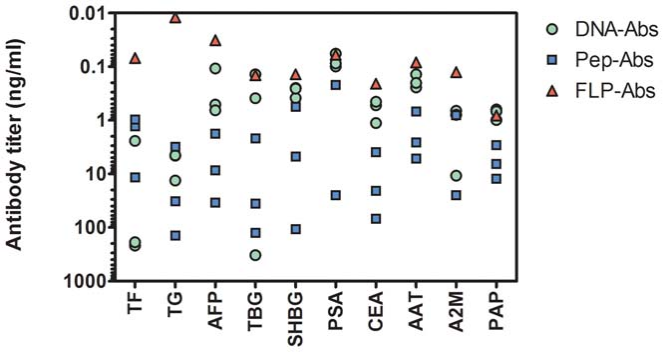
Specific activity of purified antibodies in direct bind ELISA to full length antigen. All purified antibodies were titered against full length antigen in direct bind ELISA. Data was processed with a four parameter curve fit (XLfit, IDBS, Guildford, UK) and expressed as the quantity of purified antibody required to give an absorbance of 0.5 at 650 nm using a TMB substrate. Lower amounts of antibody required (shown on an inverted scale) are indicative of higher specific activity). Each symbol represents the activity for each pool of 2 rabbits. There are 3 constructs per protein for each of the DNA-Ab and Pep-Ab methods and a single data point for the pool from full length native immunization (FLP-Abs).

In direct bind ELISAs (Figure 3), 7 of 10 DNA-Abs showed better specific activity than the respective Pep-Abs. In 7 instances (AFP, TBG, SHBG, PSA, CEA, AAT, PAP) one of the 3 DNA-Abs performed close to the value yielded by the respective FLP-Abs. In only 2 instances (SHBG, PSA) did a single Pep-Ab for each protein show a performance similar to that of respective FLP-Abs. Overall, specific activities of Pep-Abs were significantly lower (P<0.01) than those of DNA-Abs (Table 1).

**Figure 4:**
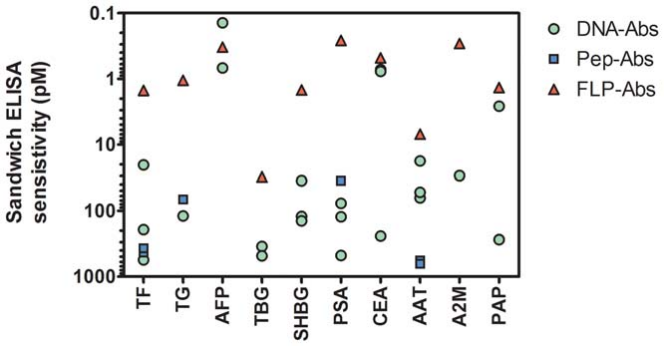
Sensitivity of antibodies in sandwich ELISA when paired with an antibody to full length protein. Standard curves were processed with four parameter curve fitting software and sensitivity expressed as the amount of antigen that could be detected at an OD 650 nm of 0.1 absorbance units above background. Each symbol represents the sensitivity for each pool of 2 rabbits. There are 3 constructs per protein for each of the DNA-Ab and Pep-Ab methods and a single data point for the pool from full length native immunization (FLP-Abs).

In sandwich ELISAs, DNA-Abs were more sensitive (lower limit of detection) than Pep-Abs, but were less sensitive than FLP-Abs (Figure 4). In sandwich assays paired with FLP-Abs, DNA-Abs showed significantly higher (P<0.01) sensitivity than Pep-Abs (Table 1). Both Pep-Abs and DNA-Abs did not achieve the sensitivity of assays configured solely with FLP-Abs.

In a number of instances, it was possible to configure a sandwich assay with 2 DNA-Abs raised against the same target, and more rarely with a single DNA-Ab (self-sandwich), suggesting multiple epitopes recognized by the polyclonal antisera. Ten, 6, and 4 of the 29 DNA-Abs could be paired to produce assays with sensitivities of at least 1 nM, 100 pM, and 10 pM, respectively. In contrast, no Pep-Ab was capable of producing a sandwich assay of 1 nM sensitivity with another Pep-Ab raised against the same protein. As would be expected due to the nature of a single epitope, no Pep-Ab would pair with itself (self-sandwich).

### Western blot analysis

A total of 69 antibodies generated by three immunization strategies were tested for immuno-reactivity against full length SDS-denatured protein by Western blot analysis (Table 2). Of the 29 DNA-Abs, 26 (90%) and 27 (93%) recognized the denatured protein at either the 100 ng/ml or 1000 ng/ml antibody probing concentration, respectively. Only 14 of 30 (47%) and 17 of 30 (57%) Pep-Abs were immuno-reactive at either concentration while all FLP-Abs performed well. Overall, the likelihood of observing an immuno-reactive antibody for Western blot applications was significantly greater for DNA-Abs than for Pep-Abs, as indicated by odds ratios for DNA-Abs vs. Pep-Abs of 9.9 (95% CI 2.5 to 39.9) and 10.3 (95% CI 2.1 to 51.5) for the lower and higher antibody probing concentrations tested, respectively (P<0.01). A 10-fold increase in the antibody probing concentration yielded positive Western blot results for 3 additional Pep-Abs and 1 additional DNA-Abs that had tested negative at the lower concentration.

**Table 2:** Western blot analysis: frequency of antibodies elicited by DNA immunization methodology or via peptide and full-length protein (native) immunizations that recognized the corresponding full length protein target run under SDS-PAGE denaturing conditions.

Primary Antibody Concentration (ng/ml)					
Protein	DNA - Abs		Pep-Abs		FLP-Abs
	100	1000	100	1000	100
**A2M**	3/3	3/3	2/3	3/3	1/1
**AAT**	3/3	3/3	2/3	2/3	1/1
**CEA**	3/3	3.3	1/3	1/3	1/1
**PSA**	3/3	3/3	1/3	1/3	1/1
**AFP**	3/3	3/3	1/3	2/3	1/1
**PAP**	2/3	3/3	1/3	2/3	1/1
**TBG**	3/3	3/3	1/3	1/3	1/1
**TF**	3/3	3/3	3/3	3/3	1/1
**TG**	0/2	0/2	1/3	1/3	1/1
**SHBG**	3/3	3/3	1/3	1/3	1/1
**Totals:**	**26/29**	**27/29**	**14/30**	**17/30**	**10/10**
	**(89.6%)**	**(93.1%)**	**(46.6%)**	**(56.6%)**	**(100.0%)**

### Immunohistochemistry (IHC)

**Figure 5:**
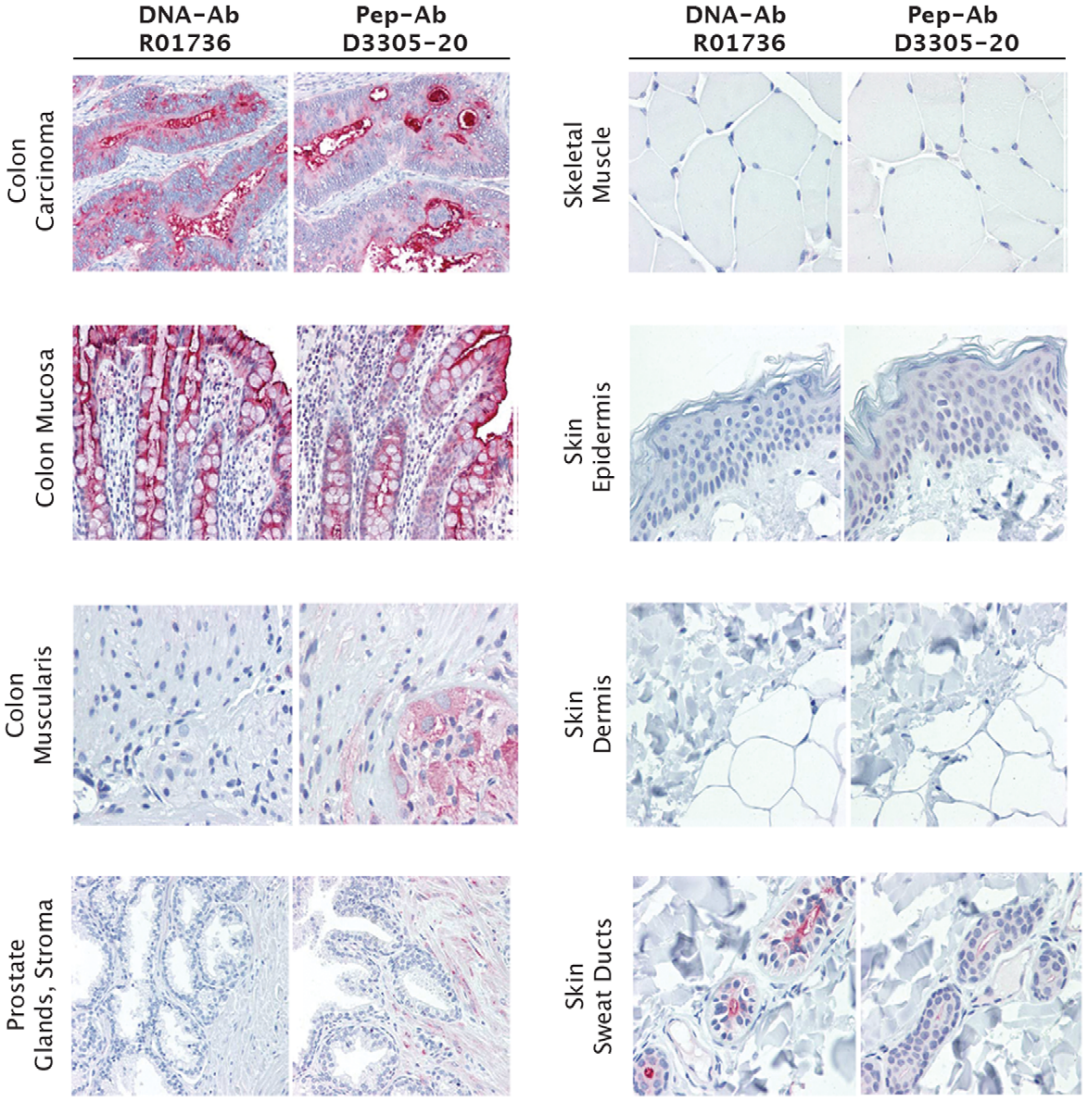
Immunohistochemical staining of human colon carcinoma and normal tissue specimens for carcinoembryonic antigen (CEA). Staining was performed with highest rank antibody R10736 and lowest rank D3305-20 generated by DNA and peptide immunization, respectively. DNA-Ab R01736 was generated against the sequence encoding amino acids 410–500. Pep-Ab D3305-20 was made from a peptide of amino acids 561–570. After full optimization both of these antibodies were found to work best at 2.5 µg/ml, the level shown here. Magnification ranges from 20× to 40×.

Independent of immunization strategy, all seven anti-CEA polyclonal antibodies were considered of excellent quality for use in IHC applications showing superb staining of multiple benign colonic epithelium and colon carcinoma specimens (Figure 5). All antibodies were titered to optimal concentration in all IHC studies described here and results discussed are at the optimal concentrations. To differentiate among antibodies in the setting of this overall high performance, ranking of antibodies was primarily based on the extent of variable staining in cell types of other tissues rather than presence and/or intensity of staining of the carcinomas. For instance, the highest-ranked antibody, R01736, a DNA-Ab, showed outstanding staining of colon carcinoma and colonic epithelium, moderate staining of sweat ducts (known to express CEA [4]) and neutrophils, but showed no staining of other normal tissues known not to express CEA, such as skin (dermis and epidermis), prostate glands and stroma, colon muscularis propria, and skeletal muscle tissues (Figure 5). In contrast, the lowest-ranked anti-CEA antibody D3305-20, a Pep-Ab, showed prominent nonselective staining in ganglion cells and peripheral nerves of the prostate and colon.

**Figure 6:**
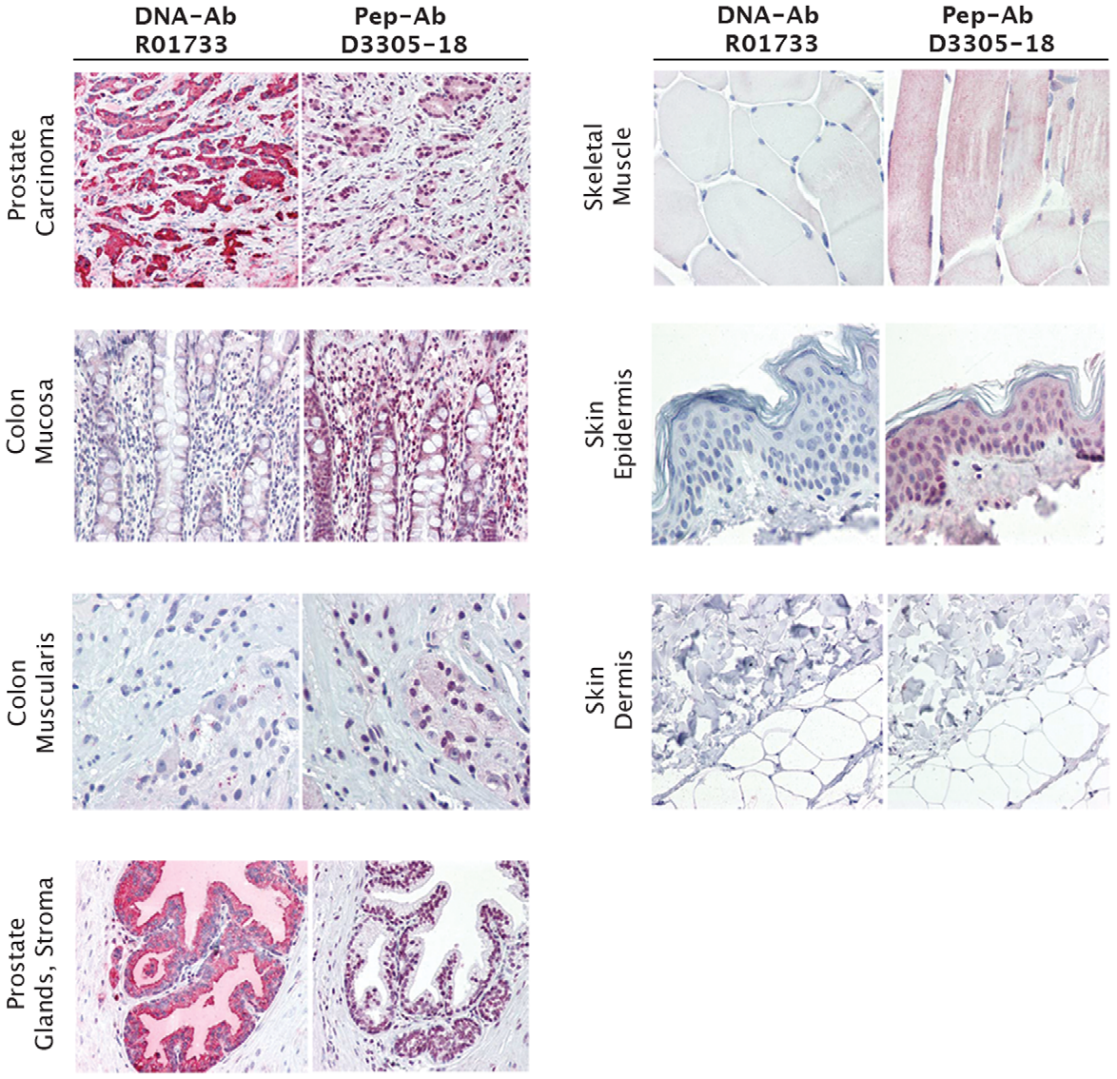
Immunohistochemical staining of human prostate carcinoma normal tissue specimens for prostate specific antigen (PSA). Staining was performed with the highest rank antibody R10733 (2.5 µg/ml) generated by DNA immunization and the lowest rank peptide-antibody D3305-18 (10 µg/ml). DNA-Ab R01733 was generated against the sequence encoding amino acids 37–139. Pep-Ab D3305-18 was made from a peptide of amino acids 126–144. Antibodies are shown at their optimized concentration, 2.5 µg/ml for R10733 and 10 µg/ml for D3305-18. Magnification ranges from 20× to 40×.

Five of 7 anti-PSA antibodies were characterized as highly successful for use in IHC showing excellent staining of prostatic epithelium and carcinoma. The highest-ranked anti-PSA antibody, R01733, was a DNA-Ab. Staining of prostate epithelium and carcinoma and differential staining of positive and negative cell types was excellent and accompanied by very little background staining (Figure 6). Staining of other tissues (skin epidermis and dermis, prostate stroma, colonic mucosa and smooth muscle, skeletal myocytes) was virtually nonexistent. In contrast, two Pep-Abs, D3305-17 and D3305-18, performed poorly in IHC. D3305-17 yielded relatively weak staining of prostatic epithelium and cancer, unimpressive differential staining of prostate in comparison with other tissues, and non-specific nuclear staining of some cell types including colon epithelium and skin (data not shown). D3305-18 showed predominantly nuclear staining in most tissues with weak to moderate positivity in colonic epithelium, smooth muscle, skin epidermis, and skeletal muscle and overall poor discrimination between positive and negative cell types (Figure 6).

### Overall success in multiple applications

Not all antibodies that worked in one application necessarily worked in other applications. Table 3 provides the cumulative success rates for each group of antibodies (Pep-Abs, DNA-Abs, FLP-Abs) across different applications. As expected, a greater fraction of Pep-Abs performed only in one application usually with a denatured target as in Western blot or IHC or in no application at all, while DNA-Abs performed more often in multiple applications, demonstrating higher versatility. The best performance under this paradigm was observed for FLP-Abs.

**Table 3:** Effect of immunization method on fitness for purpose of resultant antibodies in a number of immunologic techniques.

Immunization method			
Method	Pep-Abs	DNA-Abs	FLP-Abs
Western blot *only*	12/30 (40%)	3/29 (10%)	0/10 (0%)
IHC *only*	2/6 (33%)	1/6 (17%)	0/10 (0%)
Western blot/IHC *only*	1/6 (17%)	0/29 (0%)	0/10 (0%)
Sandwich ELISA *only*	1/30 (3%)	1/29 (3%)	0/10 (0%)
Western blot *and* sandwich ELISA <1 nM sensitivity	5/30 (17%)	22/29 (76%)	10/10 (100%)
Western blot *and* sandwich ELISA <100 pM sensitivity	1/30 (3%)	13/29 (49%)	10/10 (100%)
Western blot *and* sandwich ELISA <10 pM sensitivity	0/30 (0%)	5/29 (17%)	9/10 (90%)
Western blot *and* sandwich ELISA *and* IHC	1/6 (17%)	6/6 (1005)	6/6 (100%)
No performance in any application (i.e., complete failure)	11/30 (37%)	1/29 (3%)	0/10 (0%)

## Discussion

Despite decades of refining algorithms for optimal antigen design [18], [19], [36]–[38] the use of peptide immunization continues to suffer from poor predictability and modest overall success at generating antibodies that recognize native folded proteins, whereas success at recognizing denatured proteins can be high [15]. The performance of peptide immunization has heretofore not been compared to other approaches in a carefully designed and controlled, systematic and comprehensive study. The current investigation provides such a systematic analysis of the performance of 3 different immunization strategies, applied to 10 representative, well-characterized targets, and evaluates the respective performance of the resultant antibodies across a range of relevant assay applications. While all Pep-Abs demonstrated strong reactivity with the immunizing peptide, and while yield of affinity-purified Pep-Abs (using immobilized peptide) was acceptable, we found that 37% of Pep-Abs in this study did not perform adequately in any of the commonly used immunologic methods tested. The performance of Pep-Abs was poorest in sandwich ELISAs with native full length protein. Only 7 of 30 antibodies were capable of pairing with an antibody to full length protein to yield sensitivities of 1 nM or less, and of these only 2 demonstrated sensitivities of less than 100 pM. Pep-Abs demonstrated significantly better performance under denaturing conditions, i.e. in Western blots or IHC. At the higher concentration of probing antibody, 57% of the Pep-Abs were able to recognize the target protein in Western blots. The performance of peptide immunizations to produce affinity reagents capable of recognizing the cognate target relative to the other immunization strategies is in agreement with the rates of 25% to 50% for peptide fragments (12 to 15 residues in length) [16] and 56% (7 to 20 residues) in another studies [15] for monoclonal and polyclonal antibodies, respectively.

**Figure 7:**
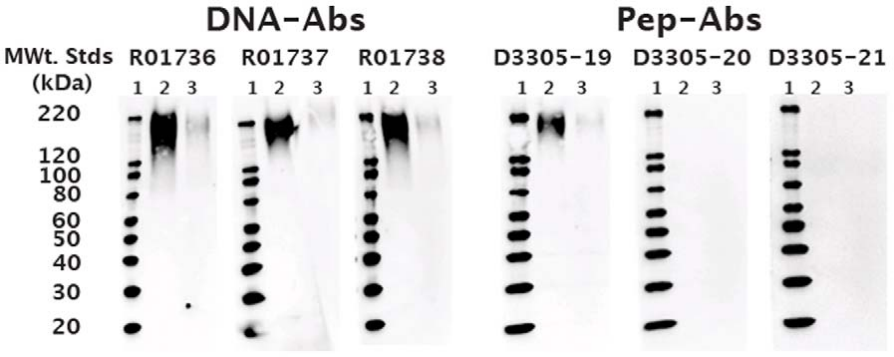
Western blot analysis of full-length carcinoembryonic antigen (CEA) run under denaturing conditions. Each immunoblot was probed with DNA (DNA-Abs) derived anti-CEA antibodies or peptide-derived (Pep-Abs) antibodies at 100 ng/ml and 1∶4000 anti-rabbit HRP. Lane 1 = molecular weight standards (kDa); Lane 2 and 3 = 10 ng and 1 ng of CEA per lane, respectively. DNA-Abs R01736, R01737, and R01738 were generated against the sequences encoding amino acids 410–500, 588–686, and 317–421 respectively. Pep-Abs D3305-19, D3305-20,and D3305-21 were made from peptides of amino acids 616–630, 561–570, and 321–339 respectively.

Even optimally designed peptides are unlikely to assume the conformational structure of the respective residues as present in the context of a full-length native, non-denatured protein. Pep-Abs are therefore expected to perform better in assays where the target protein is at least partially denatured. It is important to note that the denaturing conditions to which a protein is subjected may differ between applications, e.g. Western blotting and IHC, and that performance in one assays format may not necessarily be predictive of performance in another. Thus, 2 of the anti-CEA Pep-Abs performed well in IHC but failed to perform in Western blots under the conditions employed in this study (Figure 7), whereas no such differences were observed with anti-PSA Pep-Abs (Figure S1). Western blots for DNA-Abs and Pep-Abs for other proteins are shown in in Figures S2, S3, S4, S5, S6, S7, S8, S9.

Pep-Abs showed lower specific activity in direct-bind ELISA to native full length protein compared to DNA-Abs or FLP-Abs. It is possible that the rotational flexibility of peptide antigens leads to the induction and purification of multiple antibody species that recognize peptide conformations that do not exist in the full-length native target, thus accounting for low specific activity of affinity-purified polyclonal peptide antibody.

During the course of this study, we encountered issues that may further impact the success of commercial Pep-Abs. We observed that 26% of peptide designs provided by the peptide suppliers were predicted to be buried within the native protein's structure and therefore predicted not to be suitable for developing antibodies that recognize native structures (although such designs may well work in denaturing applications). In 9% of the designs additional issues such as high sequence identity to a paralog, presence of internal cysteine residues (that would interfere with conjugation), presence of an N-linked glycosylation site, and even deletion of an amino acid were noted that would have negatively impacted antibody performance. Thus, more than a third of the original peptide designs were *a priori* judged as not suitable for developing antibodies and were rejected, highlighting the importance of critical review of antigen design when ordering a custom Pep-Ab. It stands to reason to wonder, therefore, that in situations where the end-user has no control over peptide design (such as when ordering a premade antibody from a catalogue) suboptimal immunogen design may be of particular concern. It is possible that selecting peptide designs based on surface exposure may have introduced some bias into the success rates of the antibodies in Western blots. Peptide design is a critical variable in determining success for antibodies to recognize folded proteins. Algorithms for predicting ‘antigenicity’ and B-cell epitopes generally perform poorly [17] and not all post-translational modifications are annotated. Because of the small size of peptides, appropriate regions must be selected very carefully to ensure localization on the surface of the folded protein as well as absence of interfering post-translational modifications. We found little consensus among the three leading suppliers of peptides regarding designs, with only 14 of the 68 initial designs overlapping another company's design, and only 2 of the designs with complete consensus overlapped provided by all three suppliers. Furthermore, there were no statistically significant differences in the resulting antibody performance between the 3 companies (data not shown).

Antibodies produced by DNA-encoded polypeptide immunization draw on the advantage of being raised against a larger fraction of the protein, may potentially recognize several epitopes that are also present on the surface of the native protein, and are therefore expected to more likely react with the protein target in its correct, native conformational structure than antibodies raised using peptide antigens. The substantially higher specific activities observed for DNA-Abs compared to Pep-Abs are consistent with this argument, as are the considerably higher success rates in sandwich ELISAs where the antigens are analyzed under more physiologic, non-denaturing, conditions. Similarly, it would be expected that full length protein would encompass all the possible epitopes. Twenty-two of 29 DNA-Abs performed well in both Western blots and sandwich ELISA, and 14 of these achieved sensitivities of less than 100 pM in ELISAs using FLP-Ab as the second partner. One antibody worked in ELISA, but not Western blot, possibly indicative of highly conformation-specific properties. Only 3 of 29 antibodies worked exclusively in Western blot, but no other applications. All 6 antibodies tested in IHC showed acceptable sensitivity and tissue specificity. Only 1 DNA-Ab failed to demonstrate utility in any of the applications tested. Taken together these results suggest that the polyclonal DNA-Abs to larger polypeptide antigens contain antibodies to both linear and conformation-dependent epitopes. It is interesting to speculate whether even better results could be obtained by immunizing with a DNA construct that encompasses the entire target protein; however, the size of many of the proteins in this study, ranging from 30 kD to a 720 kD tetramer precluded the reliable use of the full length DNA; and production of the protein for boosting in E coli would prove very challenging.

**Figure 8:**
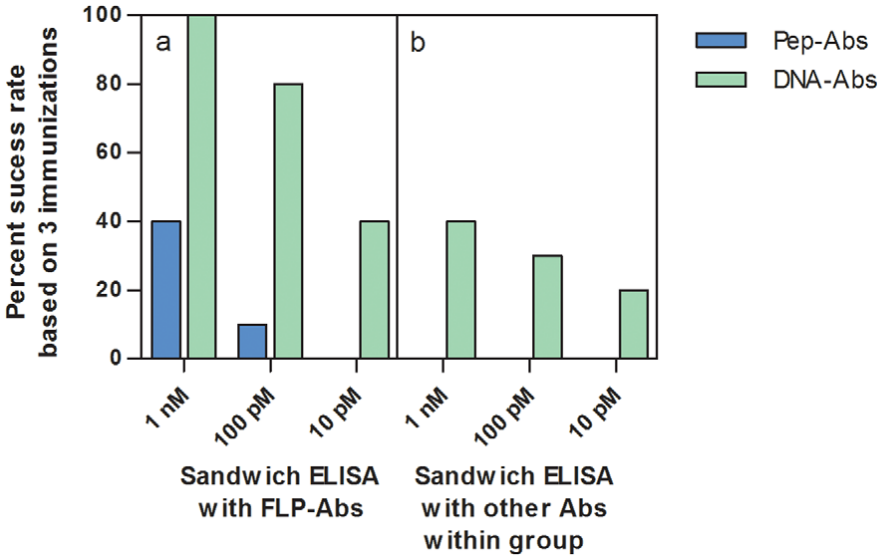
Success rates in sandwich ELISA using 3 different immunization designs for Pep-Abs or DNA-Abs. 8a) Success is defined as the percent of the time that at least one of the designs will give rise to an antibody that can pair with an FLP-Ab to the target giving sensitivity at or above the indicated concentrations. 8b) Success when at least one of the designs will form a sandwich pair with itself or with another antibody within the group (Pep-Abs with Pep-Abs, DNA-Abs with DNA Abs) to give sensitivity at or above the indicated concentrations.

Success rates across antigen strategies varied among protein targets used in the study, and it proved important to have used more than one antigen design per target. Using 3 antigen designs, we observed a 100%, 80%, and 40% chance of at least 1 of these yielding a DNA-Ab that performed in a sandwich ELISA (paired with the respective FLP-Ab) at a sensitivity of 1 nM, 100 pM, and 10 pM, respectively. Analogous likelihoods observed for Pep-Abs were 40%, 10%, and 0%, respectively (Figure 8). Success rates using not an FLP-Ab/DNA-Ab pair, but a DNA-Ab/DNA-Ab pair (typically two different DNA-Abs to the same target) in sandwich ELISAs (as might often be necessary for a new biomarker where no full length protein or FLP-Abs exist), were 40%, 30%, 20%, at sensitivities of 1 nM, 100 pM, and 10 pM, respectively. Pep-Ab/Pep-Ab-paired sandwich assays were uniformly unsuccessful at all sensitivities even when 2 different Pep-Abs to the same target were used. Similarly, for Western Blot applications, only an immunization strategy employing 3 different peptide designs ensured the recovery of at least one useful Pep-Ab for all 10 targets, even though the selected target proteins are all well established as highly immunogenic and non-challenging with regard to eliciting antibody-responses. Conversely, only one DNA-Ab antigen design would have been required for 9 of 10 target proteins given that in these 9 targets all 3 selected DNA antigens yielded DNA-Abs successful in Western blots. Only for a single protein, TG did all three of the DNA-encoded polypeptide designs fail to generate any antibody compatible with Western blot applications at any of the assay conditions tested in this study. Despite the success rate of one DNA construct design per protein within this limited sample set we recommend that multiple constructs be employed to maximize the probability of obtaining antibodies with the desired assay performance characteristics. This is certainly critical in the event of developing immunoreagents to protein biomarker targets that are immunologically more demanding than the targets addressed in the present study, and/or if antibodies with a broader scope of assay applications are intended.

It is noteworthy, that all 6 DNA-Abs tested in IHC showed excellent performance in this application. We accept that our inferences regarding the success rate in IHC applications are limited by the small number of protein targets and respective antibodies evaluated in this study. However, despite the restricted data set, the current observations confirm our previous findings and those of others that DNA-Abs perform in IHC at a level equivalent to their protein counterparts [2].

A current limitation of the DNA-based immunization approach described here relates to using a protein fragment generated in *E. coli* both for a final boost and for affinity purification. While DNA immunization has been shown to be very effective at priming immune responses, a single protein boost is recognized to often dramatically increase titers and is commonly practiced as a “prime-boost” protocol for producing antibodies [26]–[28]. Although antibody yields with this technique still appear low in comparison to Pep-Abs, their binding characteristics more than counterbalance this, thus providing higher yields if regarded from the viewpoint of specific activity. Since a prokaryotic protein is used for affinity purification, this approach would not be expected to result in purification of antibodies that recognize post-translationally modified forms of the protein, or conformational epitopes dependent on disulfide bonds. Whereas such antibodies may indeed have been generated by DNA immunization, they would have been lost during the purification step. Efforts are underway to refine the DNA immunization method by employing eukaryotically expressed protein fragments for both protein boost and in the affinity purification step. It is noteworthy, however, that use of the prokaryotic protein portion for affinity purification frequently gave rise to repertoires of antibodies that recognized denatured as well as native conformations (as evidenced by sandwich ELISA with native protein).

Immunizations with full length protein produced higher polyclonal antibody titers, greater yields, and superior performance in sandwich ELISA compared to other immunization strategies studies. These antibodies also performed well in Western blot and IHC, with a 100% success rate. The full length proteins used in this study are well-characterized serum proteins, many of which have been extensively studied for *in-vitro* diagnostic applications, and are highly immunogenic, consistent with this success rate. Their performance is likely also related to the fact that one would expect the most diverse repertoire of surface-epitope-specific antibody species with this approach.

As pointed out, immunization with full length protein is generally fraught with a number of challenges related to difficulties that may be encountered in gene construction, expression, and purification. For multiple application uses DNA-encoded polypeptide immunization may offer an effective alternative with significantly higher probabilities of success than peptide-based immunization approaches. It remains important, still, to use more than one antigen design for a given target to achieve a high probability of success for any one desired application.

An important trend in the last decade has been the development of proteome-scale studies enabling a deeper understanding of biology and a much wider search for biomarkers. The increased scale of these whole proteome-screening technologies makes the generation of high quality affinity reagents an ever-more urgent need, as these reagents are a mainstay of quantitative biological measurements. Ironically, the technology for generating what continues to be the gold standard affinity reagents, animal-immunization-derived antibodies, has lagged behind. Only a sound understanding of the attributes of antibodies generated by different approaches, and advances in the generation of such reagents applicable in proteome-wide analytical methodology will allow the field to move forward successfully. Whereas it is always critical to test antibodies in their ultimate intended application to optimize chances of success, the present study demonstrates that DNA immunization-based technology can generate antibodies that have a relatively high success rate in multiple immunoassay formats and avoid many of the limitations of Pep-Abs as well as of the challenges of FLP-Abs, thus representing a technology that can augment the arsenal of methods to fill the needs of proteomic-scale investigations. Our current study was limited to interrogate these aspects with regard to well characterized and highly immunogenic targets; additional work will be required to characterize the performance of this technology in comparison with others with regard to less immunogenic targets.

## Supporting Information

Figure S1 **Western blot analysis of full-length prostate specific antigen (PSA) run under denaturing conditions.** Each immunoblot was probed with DNA (DNA-Abs) derived anti-PSA antibodies or peptide-derived (Pep-Abs) antibodies at 100 ng/ml and 1∶4000 anti-rabbit HRP. Lane 1 = molecular weight standards (kDa); Lane 2 and 3 = 10 ng and 1 ng of PSA per lane, respectively.
(TIF)

Figure S2 **Western blot analysis of full-length prostatic acid phosphatase (PAP) run under denaturing conditions.** Each immunoblot was probed with DNA (DNA-Abs) derived anti-PAP antibodies or peptide-derived (Pep-Abs) antibodies at 100 ng/ml and 1∶4000 anti-rabbit HRP. Lane 1 = molecular weight standards (kDa); Lane 2 and 3 = 10 ng and 1 ng of PAP per lane, respectively.
(TIF)

Figure S3 **Western blot analysis of full-length thyroxine binding globulin (TBG) run under denaturing conditions.** Each immunoblot was probed with DNA (DNA-Abs) derived anti-TBG antibodies or peptide-derived (Pep-Abs) antibodies at 100 ng/ml and 1∶4000 anti-rabbit HRP. Lane 1 = molecular weight standards (kDa); Lane 2 and 3 = 10 ng and 1 ng of TBG per lane, respectively.
(TIF)

Figure S4 **Western blot analysis of full-length transferring (TF) run under denaturing conditions.** Each immunoblot was probed with DNA (DNA-Abs) derived anti-TF antibodies or peptide-derived (Pep-Abs) antibodies at 100 ng/ml and 1∶4000 anti-rabbit HRP. Lane 1 = molecular weight standards (kDa); Lane 2 and 3 = 10 ng and 1 ng of TF per lane, respectively.
(TIF)

Figure S5 **Western blot analysis of full-length thyroglobulin (TG) run under denaturing conditions.** Each immunoblot was probed with DNA (DNA-Abs) derived anti-TG antibodies or peptide-derived (Pep-Abs) antibodies at 100 ng/ml and 1∶4000 anti-rabbit HRP. Lane 1 = molecular weight standards (kDa); Lane 2 and 3 = 10 ng and 1 ng of TG per lane, respectively.
(TIF)

Figure S6 **Western blot analysis of full-length alpha-1-antitrypsin (AAT) run under denaturing conditions.** Each immunoblot was probed with DNA (DNA-Abs) derived anti-AAT antibodies or peptide-derived (Pep-Abs) antibodies at 100 ng/ml and 1∶4000 anti-rabbit HRP. Lane 1 = molecular weight standards (kDa); Lane 2 and 3 = 10 ng and 1 ng of AAT per lane, respectively.
(TIF)

Figure S7 **Western blot analysis of full-length alpha-fetoprotein (AFP) run under denaturing conditions.** Each immunoblot was probed with DNA (DNA-Abs) derived anti-AFP antibodies or peptide-derived (Pep-Abs) antibodies at 100 ng/ml and 1∶4000 anti-rabbit HRP. Lane 1 = molecular weight standards (kDa); Lane 2 and 3 = 10 ng and 1 ng of AFP per lane, respectively.
(TIF)

Figure S8 **Western blot analysis of full-length alpha-2-macroglobulin (A2M) run under denaturing conditions.** Each immunoblot was probed with DNA (DNA-Abs) derived anti-A2M antibodies or peptide-derived (Pep-Abs) antibodies at 100 ng/ml and 1∶4000 anti-rabbit HRP. Lane 1 = molecular weight standards (kDa); Lane 2 and 3 = 10 ng and 1 ng of A2M per lane, respectively.
(TIF)

Figure S9 **Western blot analysis of full-length sex hormone binding globulin (SHBG) run under denaturing conditions.** Each immunoblot was probed with DNA (DNA-Abs) derived anti-SHBG antibodies or peptide-derived (Pep-Abs) antibodies at 100 ng/ml and 1∶4000 anti-rabbit HRP. Lane 1 = molecular weight standards (kDa); Lane 2 and 3 = 10 ng and 1 ng of SHBG per lane, respectively.
(TIF)
