# Supplementary figures and images for: Impact of Immunization Technology and Assay Application on Antibody Performance – A Systematic Comparative Evaluation
Source: PLoS One. 2011 Dec 20;6(12):e28718. doi: 10.1371/journal.pone.0028718 (PMC3243671; doi:10.1371/journal.pone.0028718)

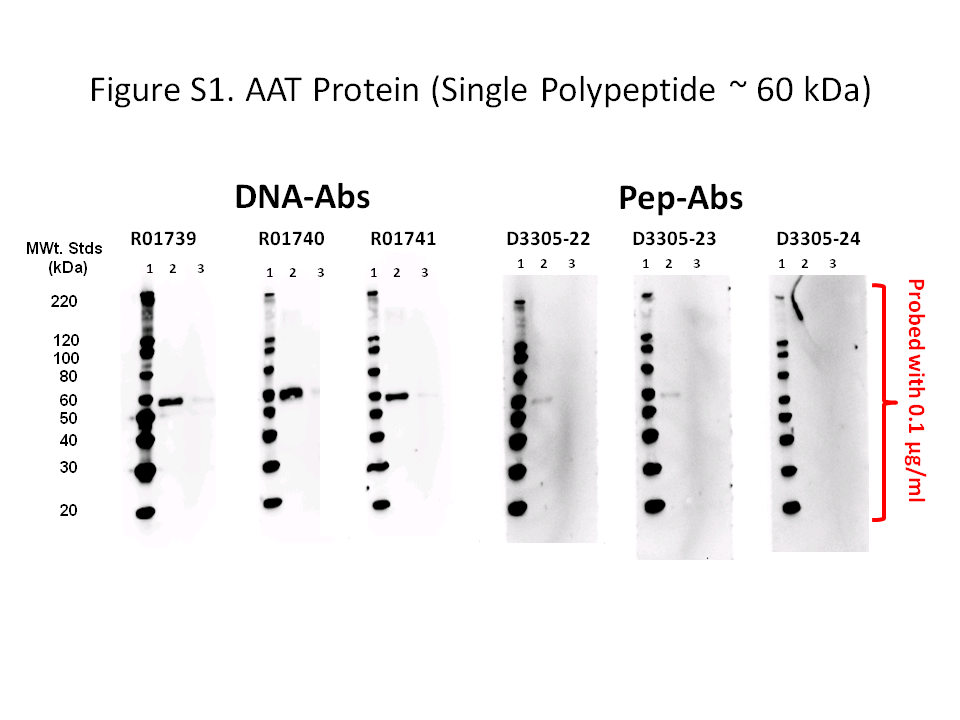

Supplement: Figure S1 — Western blot analysis of full-length prostate specific antigen (PSA) run under denaturing conditions. Each immunoblot was probed with DNA (DNA-Abs) derived anti-PSA antibodies or peptide-derived (Pep-Abs) antibodies at 100 ng/ml and 1∶4000 anti-rabbit HRP. Lane 1 = molecular weight standards (kDa); Lane 2 and 3 = 10 ng and 1 ng of PSA per lane, respectively. (TIF) [file pone.0028718.s001.tif]

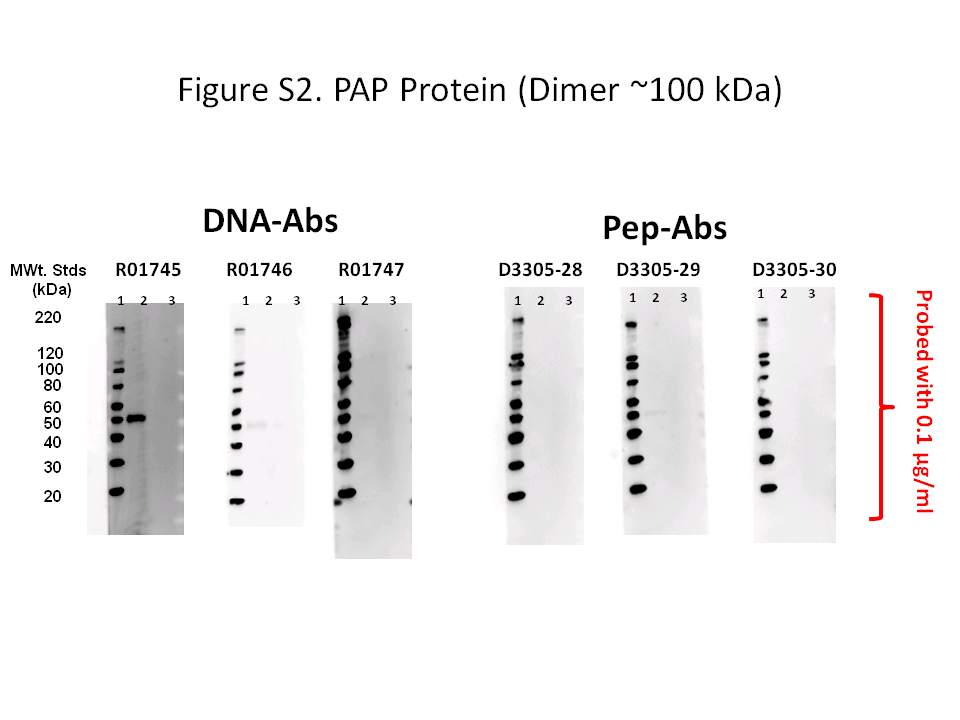

Supplement: Figure S2 — Western blot analysis of full-length prostatic acid phosphatase (PAP) run under denaturing conditions. Each immunoblot was probed with DNA (DNA-Abs) derived anti-PAP antibodies or peptide-derived (Pep-Abs) antibodies at 100 ng/ml and 1∶4000 anti-rabbit HRP. Lane 1 = molecular weight standards (kDa); Lane 2 and 3 = 10 ng and 1 ng of PAP per lane, respectively. (TIF) [file pone.0028718.s002.tif]

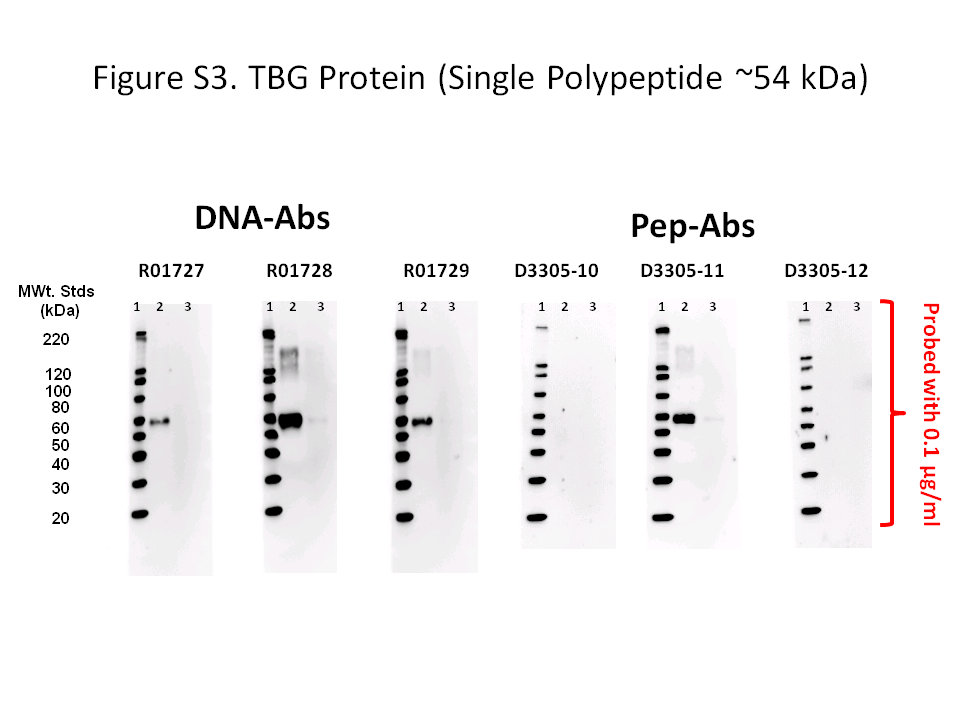

Supplement: Figure S3 — Western blot analysis of full-length thyroxine binding globulin (TBG) run under denaturing conditions. Each immunoblot was probed with DNA (DNA-Abs) derived anti-TBG antibodies or peptide-derived (Pep-Abs) antibodies at 100 ng/ml and 1∶4000 anti-rabbit HRP. Lane 1 = molecular weight standards (kDa); Lane 2 and 3 = 10 ng and 1 ng of TBG per lane, respectively. (TIF) [file pone.0028718.s003.tif]

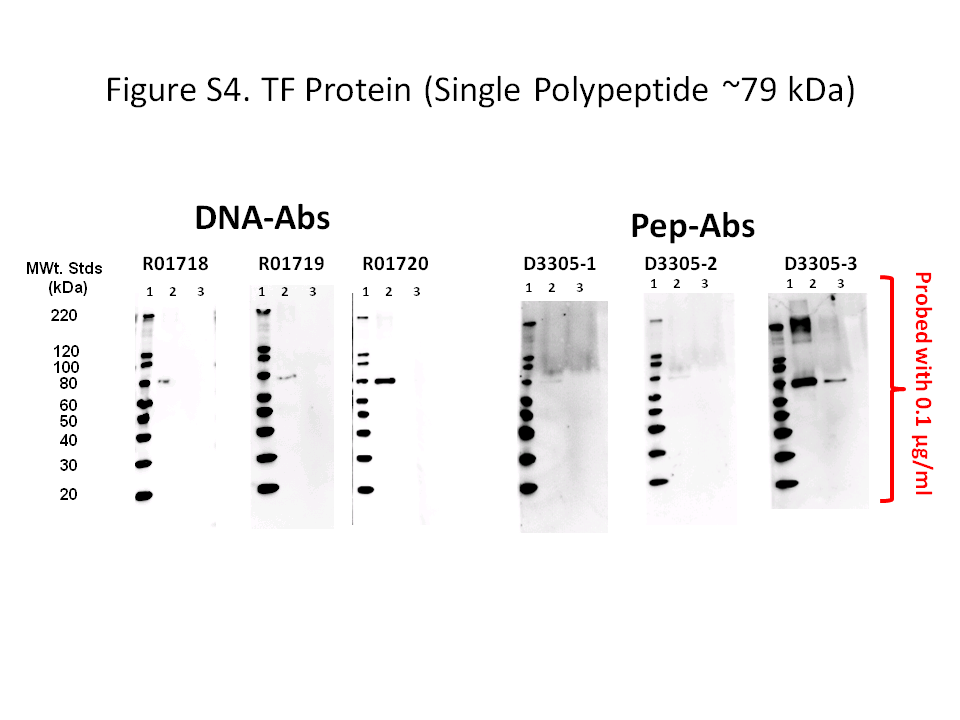

Supplement: Figure S4 — Western blot analysis of full-length transferring (TF) run under denaturing conditions. Each immunoblot was probed with DNA (DNA-Abs) derived anti-TF antibodies or peptide-derived (Pep-Abs) antibodies at 100 ng/ml and 1∶4000 anti-rabbit HRP. Lane 1 = molecular weight standards (kDa); Lane 2 and 3 = 10 ng and 1 ng of TF per lane, respectively. (TIF) [file pone.0028718.s004.tif]

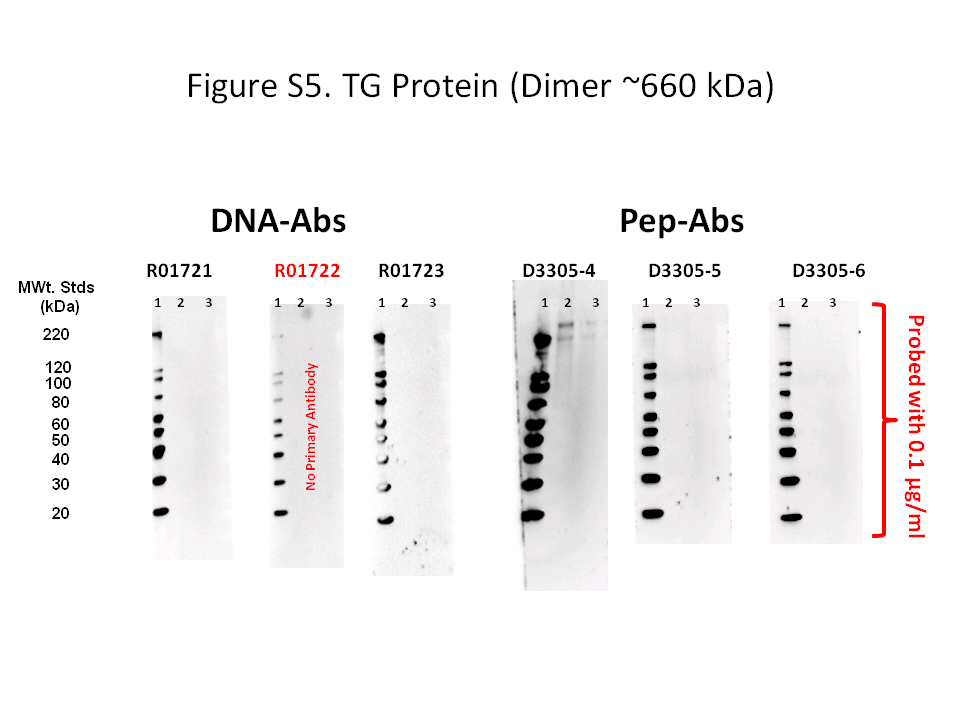

Supplement: Figure S5 — Western blot analysis of full-length thyroglobulin (TG) run under denaturing conditions. Each immunoblot was probed with DNA (DNA-Abs) derived anti-TG antibodies or peptide-derived (Pep-Abs) antibodies at 100 ng/ml and 1∶4000 anti-rabbit HRP. Lane 1 = molecular weight standards (kDa); Lane 2 and 3 = 10 ng and 1 ng of TG per lane, respectively. (TIF) [file pone.0028718.s005.tif]

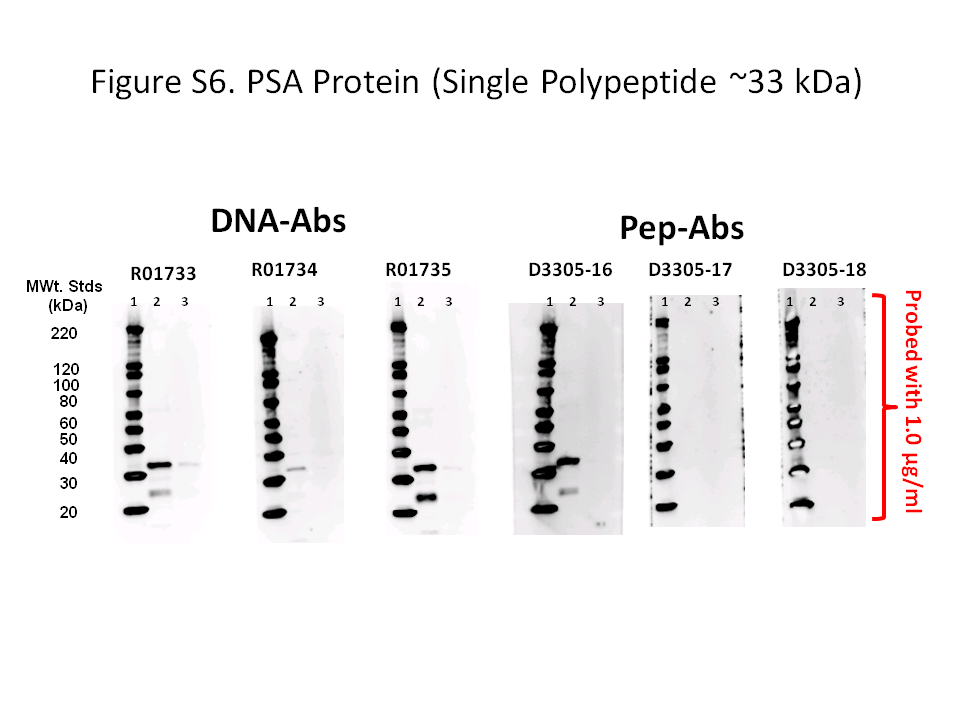

Supplement: Figure S6 — Western blot analysis of full-length alpha-1-antitrypsin (AAT) run under denaturing conditions. Each immunoblot was probed with DNA (DNA-Abs) derived anti-AAT antibodies or peptide-derived (Pep-Abs) antibodies at 100 ng/ml and 1∶4000 anti-rabbit HRP. Lane 1 = molecular weight standards (kDa); Lane 2 and 3 = 10 ng and 1 ng of AAT per lane, respectively. (TIF) [file pone.0028718.s006.tif]

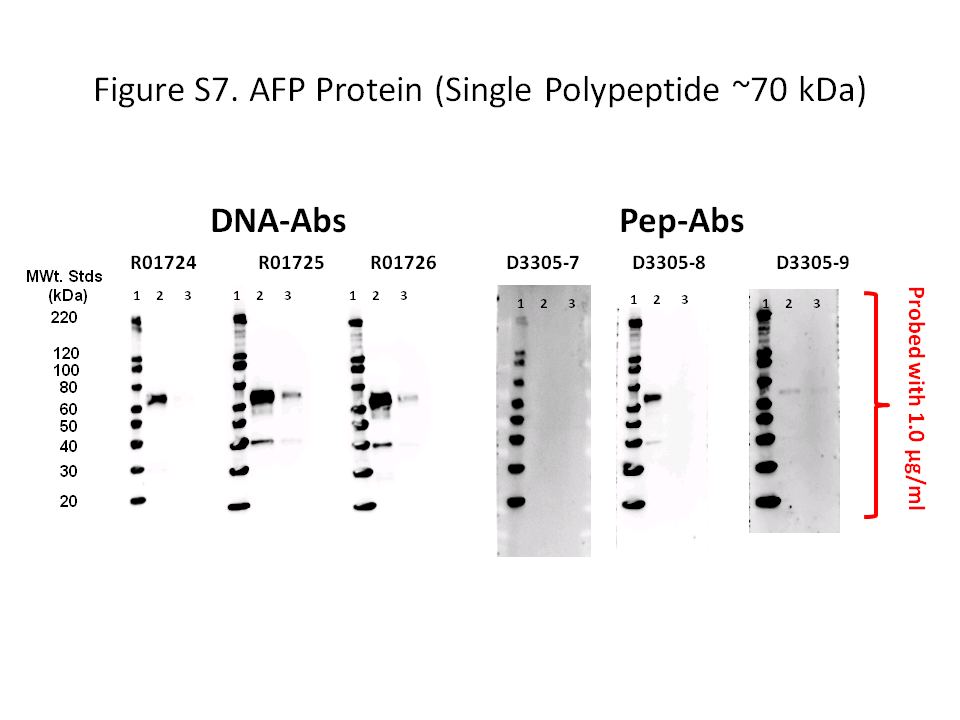

Supplement: Figure S7 — Western blot analysis of full-length alpha-fetoprotein (AFP) run under denaturing conditions. Each immunoblot was probed with DNA (DNA-Abs) derived anti-AFP antibodies or peptide-derived (Pep-Abs) antibodies at 100 ng/ml and 1∶4000 anti-rabbit HRP. Lane 1 = molecular weight standards (kDa); Lane 2 and 3 = 10 ng and 1 ng of AFP per lane, respectively. (TIF) [file pone.0028718.s007.tif]

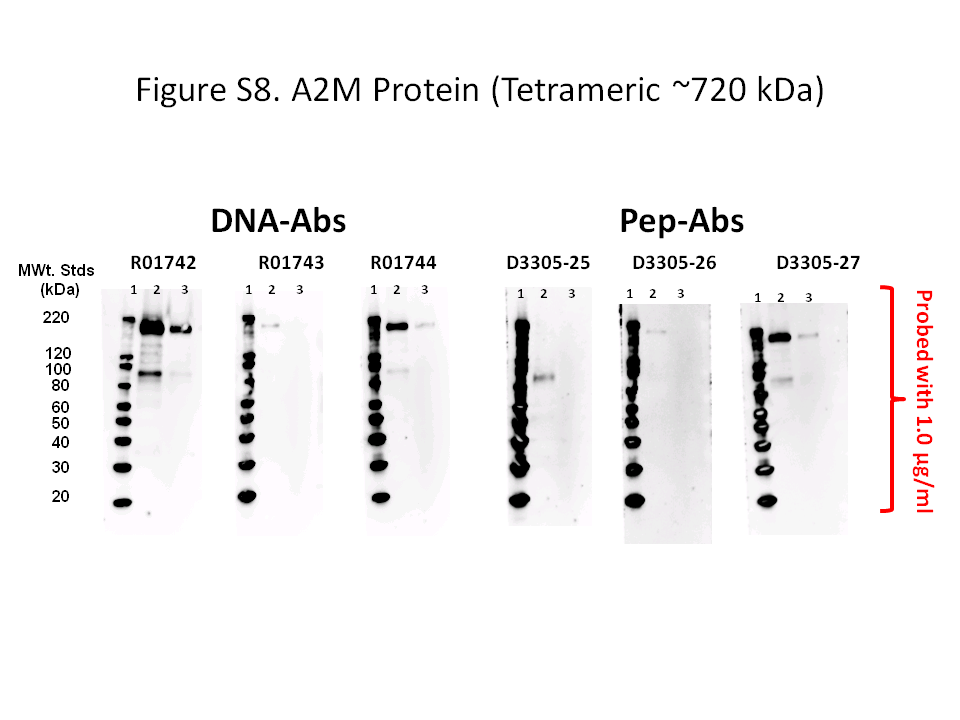

Supplement: Figure S8 — Western blot analysis of full-length alpha-2-macroglobulin (A2M) run under denaturing conditions. Each immunoblot was probed with DNA (DNA-Abs) derived anti-A2M antibodies or peptide-derived (Pep-Abs) antibodies at 100 ng/ml and 1∶4000 anti-rabbit HRP. Lane 1 = molecular weight standards (kDa); Lane 2 and 3 = 10 ng and 1 ng of A2M per lane, respectively. (TIF) [file pone.0028718.s008.tif]

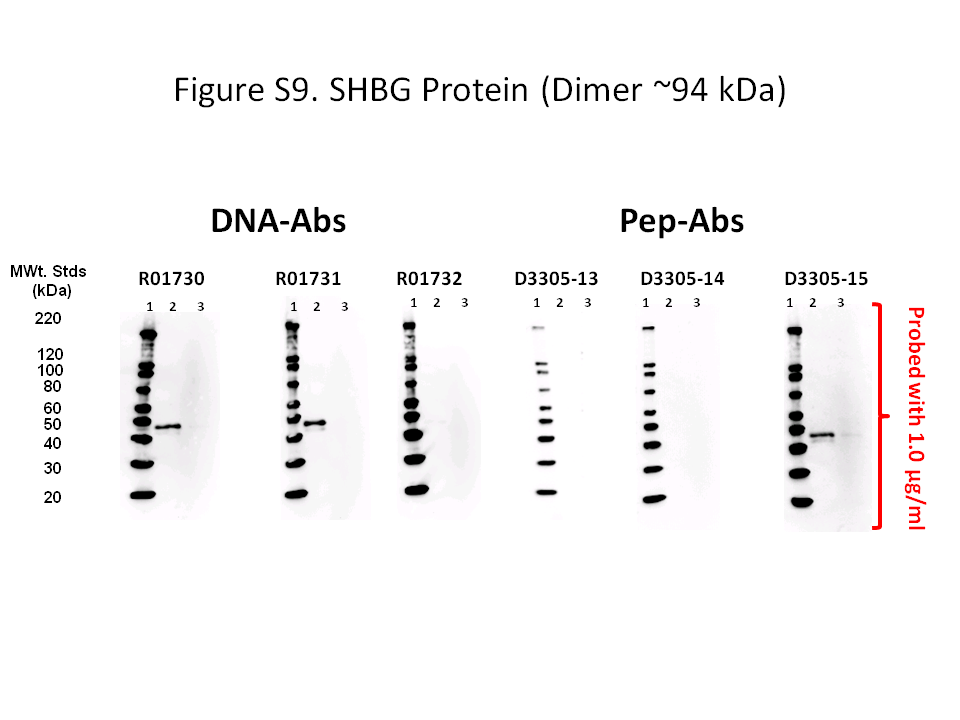

Supplement: Figure S9 — Western blot analysis of full-length sex hormone binding globulin (SHBG) run under denaturing conditions. Each immunoblot was probed with DNA (DNA-Abs) derived anti-SHBG antibodies or peptide-derived (Pep-Abs) antibodies at 100 ng/ml and 1∶4000 anti-rabbit HRP. Lane 1 = molecular weight standards (kDa); Lane 2 and 3 = 10 ng and 1 ng of SHBG per lane, respectively. (TIF) [file pone.0028718.s009.tif]
